# Acquisition and Analysis of Microcirculation Image in Septic Model Rats

**DOI:** 10.3390/s22218471

**Published:** 2022-11-03

**Authors:** Chen Ye, Mami Kawasaki, Kazuya Nakano, Takashi Ohnishi, Eizo Watanabe, Shigeto Oda, Taka-Aki Nakada, Hideaki Haneishi

**Affiliations:** 1 Center for Frontier Medical Engineering, Chiba University, Chiba 263-8522, Japan; 2 Graduate School of Science and Engineering, Chiba University, Chiba 263-8522, Japan; 3 Faculty of Science and Technology, Seikei University, Tokyo 180-8633, Japan; 4 Memorial Sloan Kettering Cancer Center, New York, NY 10065, USA; 5 Department of Emergency and Critical Care Medicine, Graduate School of Medicine, Chiba University, Chiba 263-8522, Japan

**Keywords:** microcirculation, sepsis, thrombomodulin (TM) alfa, blood velocity, vessel diameter, robust principal component analysis (RPCA)

## Abstract

**Background**: Microcirculation is a vital sign that supplies oxygen and nutrients to maintain normal life activities. Sepsis typically influences the operation of microcirculation, which is recovered by the administration of medicine injection. **Objective**: Sepsis-induced variation and recovery of microcirculation are quantitatively detected using microcirculation images acquired by a non-contact imaging setup, which might assist the clinical diagnosis and therapy of sepsis. **Methods**: In this study, a non-contact imaging setup was first used to record images of microcirculation on the back of model rats. Specifically, the model rats were divided into three groups: (i) the sham group as a control group; (ii) the cecum ligation and puncture (CLP) group with sepsis; and (iii) the CLP+thrombomodulin (TM) group with sepsis and the application of TM alfa therapy. Furthermore, considering the sparsity of red blood cells (RBCs), the blood velocity is estimated by robust principal component analysis (RPCA) and U-net, and the blood vessel diameter is estimated by the contrast difference between the blood vessel and tissue. **Results and Effectiveness**: In the experiments, the continuous degradation of the estimated blood velocity and blood vessel diameter in the CLP group and the recovery after degradation of those in the CLP+TM group were quantitatively observed. The variation tendencies of the estimated blood velocity and blood vessel diameter in each group suggested the effects of sepsis and its corresponding therapy.

## 1. Introduction

Microcirculation is the circulation of blood in blood vessels smaller than 100 μm (diameter), where numerous substances and gases, such as oxygen and carbon dioxide, are exchanged. Sepsis results in tissue hypoxemia and organ dysfunction that disrupt microcirculation, which is caused by a dysregulated host response to infection. In the clinical scenario, sepsis-related organ dysfunction is associated with an in-hospital mortality greater than 10% [1]. Thrombomodulin (TM) is an integral membrane protein expressed on the surface of endothelial cells and serves as a cofactor for thrombin, which can reduce blood coagulation by converting thrombin to an anticoagulant enzyme from a procoagulant enzyme. Correspondingly, some medicines, including TM alfa, have been developed to treat sepsis-induced coagulopathy [2], and the effectiveness of TM alfa has been verified in septic model rats by blood analysis [3,4]. In particular, recombinant thrombomodulin was suggested to be administered to patients with sepsis-associated disseminated intravascular coagulation (DIC) in a recent report [5].

However, blood analysis does not provide spatiotemporal information on microcirculation. The appearance of microcirculation, velocity of blood flow, and distribution of blood clots are significant factors in understanding the condition of microcirculation and the effects of medicine [6]. Some optical setups for imaging human microcirculation [7,8,9,10] have been proposed, and much research on microcirculation image analysis has been reported [11,12,13,14,15,16,17,18,19]. However, to the best of our knowledge, there is no report of an image-based thorough investigation of the microcirculation in septic conditions and after therapy with TM alfa. Thus, we performed this investigation using model rats. To this end, we built a non-contact imaging setup that can avoid disturbance by contact imaging and obtain high-definition motion pictures. We also proposed an image analysis method to estimate the red blood cells (RBCs) velocity and blood vessel diameters.

In this paper, model rats were created by the cecum ligation and puncture (CLP) procedure because ligation and puncture on the cecum usually cause bacterial peritonitis and eventually result in sepsis [20]. For the acquisition of microcirculation images, the model rats were classified into three groups. The first one is the cecum ligation and puncture (CLP) group, wherein the rats are surgically operated on using CLP [20]. The second group was termed the CLP+TM group, wherein rats were treated with TM alfa [3,4] after CLP. The third group was termed the sham group, wherein the rats were not operated on by CLP or injected with TM alfa. Here, the sham group is deemed as the benchmark, and the effects of sepsis and TM alfa-induced therapy can be detected by the CLP group and the CLP+TM group, respectively.

Moreover, to investigate the effects of sepsis and therapy on microcirculation, a novel image analysis method comprising two approaches was proposed to estimate blood velocity and blood vessel diameter. In the estimation of blood velocity, robust principal component analysis (RPCA) [21,22] was adopted to localize the RBCs existing in blood vessels, and U-net [23] was adopted to segment the blood vessel area. In addition, the difference in pixel values between the regions of the blood vessel and tissue was calculated to estimate the blood vessel diameters. Our preliminary work has been presented in [24].

The two main contributions of this paper are summarized as follows:

1. To investigate the two indices of microcirculation under sepsis and therapy, namely, the velocity and vessel diameter of blood, two corresponding image-based estimation approaches have been proposed.
2. By observing the temporal changes in blood velocity and vessel diameter, the effects of disease and TM alfa-induced therapy on sepsis have been confirmed using multiple indices.

The remainder of this paper is organized as follows. Section 2 presents the non-contact imaging setup used in this study. Section 3 details the proposed image-analysis method. The experimental results and a discussion are respectively presented in Section 4 and Section 5, and the conclusions are provided in Section 6.

## 2. Non-Contact Imaging Setup

**Figure 1:**
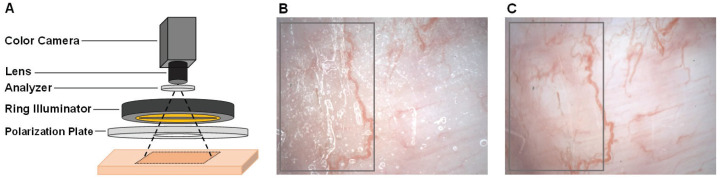
Non-contact imaging setup using a 14-bit color camera. (**A**) An illustration of the improved non-contact imaging setup. (**B**) An example of the acquired image without a polarization plate. (**C**) An example of the acquired image with a polarization plate.

Figure 1A shows the non-contact imaging setup without an invasion, which avoids the influence on blood circulation. The imaging setup was assembled using a 14-bit color camera (GS3-U3-15S5C-C, image size: 1384 × 1032 pixels, FLIR Systems Inc., Wilsonville, OR, USA) with a lens (No. 88-354, work distance: 13.5 mm, Edmund Optics Inc., Barrington, NJ, USA), common round-shaped analyzer, ring illuminator (IOTR-80-25RLGB, Shimatec Co., Ltd., Tokyo, Japan), and polarization plate (USP-50C0.4-38, SIGMAKOKI Co., Ltd., Tokyo, Japan). In the imaging setup, the rounded analyzer was set under the lens and the polarization plate was set under the ring illuminator. The ring illuminator consisted of three-color light-emitting diodes (LEDs), and the peaks of wavelength of each LED were 639 nm, 525 nm, and 461 nm, respectively, for red, green, and blue (RGB). In the geometry, the area in the object space corresponding to each pixel in the image space approximately reaches 0.64 × 0.64 μm${}^{2}$. Motion pictures of the microcirculation of model rats can be observed and acquired using this non-contact imaging setup.

In particular, to reduce the specular reflection from the tissue surface of rats, a polarization plate was placed as crossed Nicols. Based on the non-contact imaging setup, the effect of the polarization plate was confirmed by comparing the acquired image examples in Figure 1B,C. Compared with the image in Figure 1B without a polarization plate, the evident reduction in specular reflection of the image in Figure 1C is confirmed, which is also highlighted by rectangles.

## 3. Proposed Image Analysis Method

**Figure 2:**
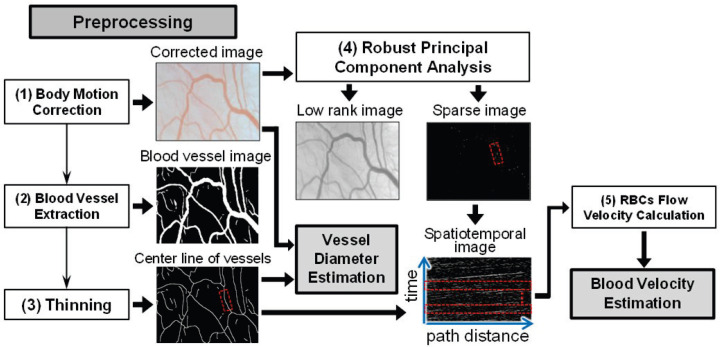
The flowchart of the proposed blood velocity estimation approach. (**1**) Body motion correction of whole acquired images is executed for the image registration. (**2**) Blood vessel extraction is operated by segmenting the corrected images based on U-net. (**3**) Thinning technique narrows down the vessels to obtain their center lines. (**4**) Robust principal component analysis (RPCA) is used to extract the red blood cells (RBCs) as the spare component. (**5**) RBCs flow velocity is calculated by the spatiotemporal image, which is constructed by the pixel values of sequential sparse component along a vascular center line.

To analyze the acquired motion pictures of microcirculation using the non-contact imaging setup, two key indices of microcirculation were estimated, namely the blood velocity and blood vessel diameter, by proposing two corresponding estimation approaches. In this section, common preprocessing is described, and the estimation approaches for blood velocity and blood vessel diameter are depicted. Figure 2 shows a general flowchart of the processing; therein, the preprocessing and RPCA can improve substantially the contrast between blood vessels and their surrounding tissue.

### 3.1. Preprocessing

For preprocessing, the body motion correction of whole images, vessel extraction, and thinning of extracted vessels were conducted. The details of this process are described below.

**Figure 3:**
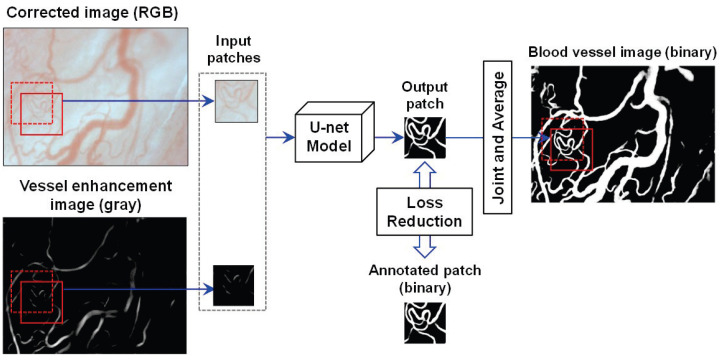
The training and prediction of the U-net model [23] for blood vessel extraction.

(1) **Body Motion Correction:** A motion correction is first executed on all the acquired images to obtain the corrected images due to the body motion-induced image blurring in the motion pictures. More concretely, the first image is defined as the target image, and each of the following images is registered to the target image. A common template-matching technique was used for this purpose.
(2) **Vessel Extraction:** The blood vessels are segmented by U-net [23] based on fully convolutional networks (FCN) [25], relying on the corrected image. The architecture of the U-net is composed of a contracted path for data information capture and a symmetric expanding path for accurate localization. In [23], U-net was proven to realize the precise and efficient segmentation of biomedical images by classifying each pixel. In the training phase, the pixel values of the vessels in the corrected images are enhanced to obtain vessel enhancement images using the enhancement filtering technique in [26]. Both the corrected images and the obtained vessel enhancement images were used to acquire a learning model that can generate images of blood vessels.
  Figure 3 illustrates the U-net model training and prediction for blood vessel extraction. To better segment the vessel fields, both the corrected image and its vessel enhancement image are used as input images. Here, the corrected image is an RGB one with three channels, and the enhancement image is a gray one with one channel. To simplify the segmentation task, the patches in the same field of the corrected and enhancement images are input simultaneously into the U-net model with overlaps to other patches for seamless segmentation, as in [23]. A binary patch from the annotated image is used to calculate and reduce the loss with the updating output patch for training the model. Using the trained U-net model, the binary vessel patches can be predicted, which are finally joined into an entire vessel image by averaging overlaps.
(3) **Thinning:** After vessel extraction based on the U-net, the thinning technique presented in [27] narrows down the regions of the vessel in the blood vessel image to obtain the center line of the vessels.

### 3.2. Estimation Approach of Blood Velocity

In our proposed blood velocity estimation approach, the velocity of the RBCs flow existing in blood vessels is regarded as that of blood flow, as in [16,18]. Figure 2 shows a flowchart of the proposed estimation approach, which consists of the following two main portions (see Blocks (4) and (5) in Figure 2).

(1) **RPCA:** Robust principal component analysis (RPCA) can perform singular value decomposition (SVD)-based dimensionality reduction, similar to standard PCA, and extract the sparse component using the introduced sparse penalty [21,22]. Thus, RPCA was adopted on the corrected image to extract the RBCs existing in the blood vessels. By RPCA, the obtained low-rank component of tissue and vessels contains elements with less temporal changes, whereas the extracted sparse component of RBCs and residual body motion noise contain elements with obvious temporal changes. The resultant image composed of the sparse component is called the sparse image in this study and is used for the calculation of the RBCs flow velocity, that is, blood velocity.
(2) **RBCs Flow Velocity Calculation:** Finally, the spatiotemporal image is constructed by the pixel values of the sequential sparse component (in the sparse images) along the center line of the vessel, and the slope of the obvious moving tracks of RBCs on the spatiotemporal images is calculated as the blood velocity.

**Figure 4:**
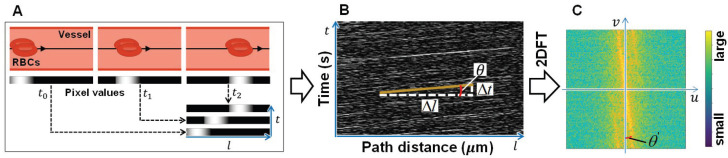
The calculation of RBCs flow velocity. (**A**) Pixel values of sequential sparse component along center line of the vessel. (**B**) Spatiotemporal image. (**C**) Intensity image of Fourier spectra (frequency domain).

Figure 4 illustrates the calculation of the RBCs flow velocity. To construct the spatiotemporal image, the pixel values of the sparse image along a certain section of the vessel center line were arranged from the time-step of $t_{0}$ in a frame order. As the sparse image mainly contains RBCs, the flow of the RBCs naturally forms white oblique lines in the spatiotemporal image. The velocity of RBCs flow can be calculated using the slope of the path length change and a short time change, as shown in Figure 4B. Here, *l* denotes the flow length of RBCs during a short period of *t*. Using the included angle θ between a horizontal line and an RBCs-induced oblique line, the quotient of *l* and *t* can also be represented by cotθ.

To obtain a more reliable velocity of RBCs flow, the intensity image of Fourier spectra (frequency domain) to a square spatiotemporal image was obtained by two-dimensional Fourier transformation (2DFT) (see Figure 4C). As an arbitrary image can be constructed by the sum of planar waves with various frequencies at multiple angles, each pixel of the intensity image in Figure 4C saves the information of each planar wave, where the axes of *u* and *v*, respectively, denote the frequencies of those planar waves along with the path distance *l* and the time *t* in Figure 4B. More concretely, each coordinate in the intensity image $\left[u,v\right]$ represents the normal vector of each planar wave, and the modulus $\sqrt{u^{2}+v^{2}}$ and direction of the normal vector represent the frequency and propagation direction of the corresponding planar wave, respectively. Meanwhile, a larger intensity of one pixel implies that the corresponding planar wave occupies more components in the spatiotemporal image, as shown in Figure 4B.

We found that the dominant yellow line in the intensity image in Figure 4C is orthogonal to the white oblique lines in the spatiotemporal image. The dominant line in low spatial frequencies where $\sqrt{u^{2}+v^{2}}$ is relatively small has larger intensities, which is focused on the calculation of the RBCs flow velocity. By defining $\theta^{{}^{\prime }}$ as the included angle between a vertical line (*v* axis) and the dominant line, which equals the average θ, $\cot\theta^{{}^{\prime }}$ is calculated as the velocity of the RBCs flow. By observing experimentally, approximately 150 sequential images are necessary for always obtaining a dominant yellow line in the intensity image in different scenarios to estimate an accurate RBCs flow velocity.

### 3.3. Estimation Approach of Blood Vessel Diameter

**Figure 5:**
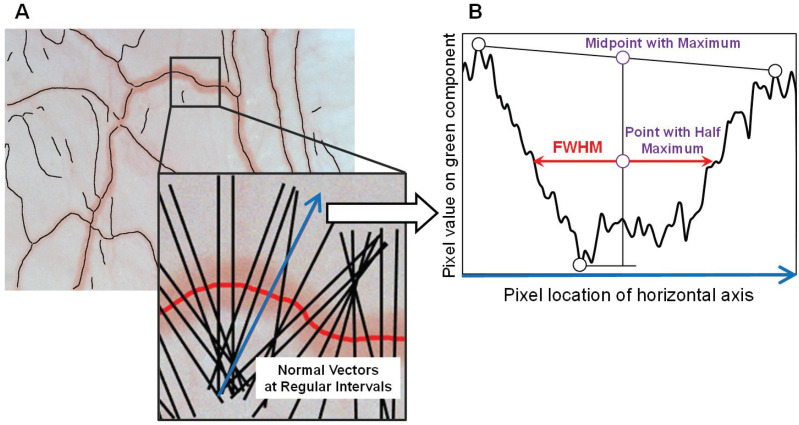
The estimation of blood vessel diameter (**A**) Overlay of corrected image and center line of vessels. (**B**) Pixel value profile in horizontal axis.

In addition to blood velocity, blood vessel diameter is another important index of microcirculation. The estimation procedure is shown in Figure 5.

In view of the distinct difference in contrast between the blood vessel region and tissue region, first, normal vectors vertical to the center line of the blood vessel are created at regular intervals. Because green illumination is more easily absorbed by hemoglobin, the pixel values of the tissue field are typically larger than those of the vessel field in a green channel image with a large contrast [28]. Subsequently, the pixel value profile of the green component of the captured color image along a normal vector direction was calculated, and the full width at half maximum (FWHM) of the distribution of pixel values was calculated as the blood vessel diameter.

As shown in Figure 5B, the direction of a selected normal vector was first converted to the horizontal axis in the chart. Next, the midpoint of the highest peaks of pixel values on each side of a blood vessel was calculated, and the difference in the pixel values between the minimum and the midpoint was regarded as the maximum. Finally, the length of the horizontal line segment through the point with the half maximum was obtained as the FWHM.

## 4. Experiments

In this section, the experimental dataset and condition for microcirculation observation, the blood vessel extraction by U-net [23], and the results consisting of measurements of the four vital signs and quantitative estimations of the blood velocity and blood vessel diameter are presented.

### 4.1. Dataset and Condition

Observation experiments on the microcirculation of model rats were performed to investigate the effects of sepsis and TM alfa. These experiments were approved by the Institutional Animal Care and Use Committee of Chiba University (No. 2-46). Table 1 lists the properties of the septic model rats. A total of 15 male Wistar rats aged 12 weeks with body weights of 240–290 g were selected as subjects. To compare the differences in microcirculation in the three distinct groups, namely sham, CLP, and CLP+TM, 15 rats were randomly and evenly divided into three groups (5 rats in each group). Here, the sham group was deemed as a control group, the CLP group was used for detecting the effect of sepsis, and the CLP+TM group was used for detecting the therapy by TM alfa. To diminish the factors that may impact blood vessels, we strictly kept the ambient temperature (24 degrees Celsius) termed as a usual setting throughout the whole experiment. Meanwhile, the relatively straight vessels were selected to estimate the blood velocity and the vessel diameter.

**Figure 6:**
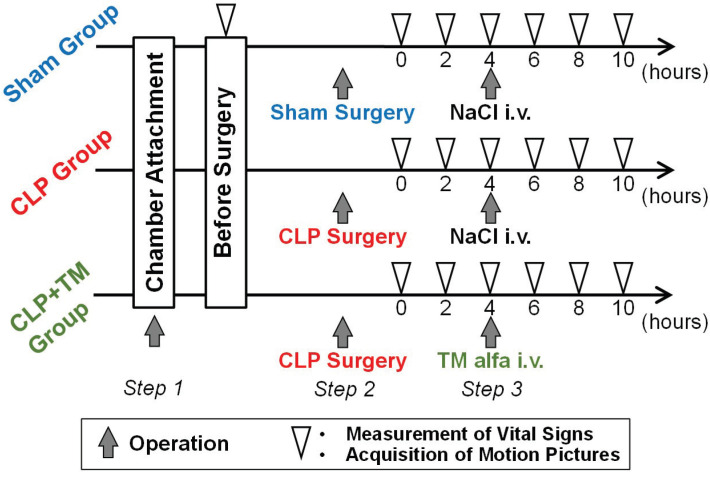
The experimental protocol of the three groups of model rats.

**Figure 7:**
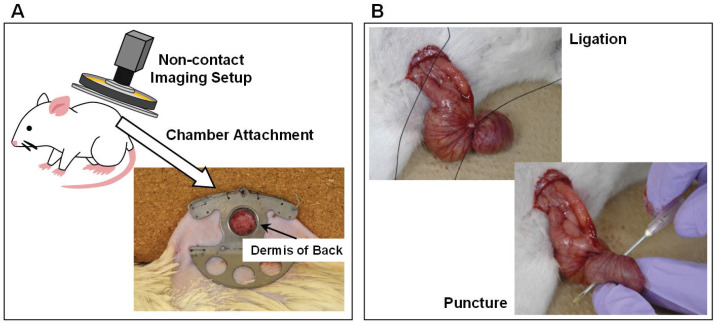
Operations and vital signs measurements in the experiments (**A**) Operation of chamber attachment. (**B**) Operation of cecum ligation and puncture (CLP).

The experimental protocol for the three groups is shown in Figure 6, which aims at detecting the temporal change caused by sepsis and its therapy using microcirculatory images. The operations were performed in three steps. In *Step 1,* for the preparation of the microcirculation observation, a dorsal window chamber was placed on the dermis of a rat’s back to flatten the dermal surface instead of a cover glass (see Figure 7A). In the experiments, we manually fine-tuned the distance between the lens and a rat’s back in the image acquisition to obtain a focused image and ensured that the acquired images could always cover each targeted section of blood vessels. Note that the manual distance fine-tuning could be easily and rapidly conducted for obtaining focused images by the adopted non-contact imaging setup. In addition, it was not frequently conducted, since hundreds of sequential focused images could be obtained after each manual fine-tuning. The typical exposure time and gain are 21.81 ms and 10.14 dB, respectively. In *Step 2,* a sham surgery was conducted on the rats in the sham group, which only contained a laparotomy with a suture. In contrast, ligation and puncture, termed CLP surgery, were conducted on the rats in both CLP and CLP+TM groups (see Figure 7B) between a laparotomy and a final suture. In *Step 3*, four hours after the surgeries of sham and CLP, intravenous (i.v.) injections of NaCl were administered to both sham and CLP groups as a reference. In contrast, TM alfa (3 mg/kg BW, donated by Asahi Kasei Pharma Corporation, Tokyo, Japan) was administered to the CLP+TM group. The dosage and administration timing of NaCl and TM alfa were referred to those in [3].

Accompanying the three-steps-based operations, the measurements of vital signs and the motion pictures acquisition of microcirculation were performed synchronously in advance of the surgical operations in *Step 2* as well as every two hours up to 10 h after *Step 2* (white triangle in Figure 6). The blood of a rat model was collected from the tail vein, and lactate was measured using a compact measuring instrument (LT-1730, ARKRAY, Inc., Kyoto, Japan). The other three vital signs, including SO${}_{2}$, heart rate (HR), and respiration rate (RR) were measured using a pulse oximeter (MouseOx® PLUS, STARR LifeSciences Co., Ltd., Ave Oakmont, PA, USA) that was adhered to the left forelimb. Throughout the experiment, the rats were anesthetized with 2.0% isoflurane (Escain, Pfizer, Tokyo, Japan) as a routine. More concretely, to assimilate the contents and their quantity in the model rats’ intestines, all the 15 rats fasted except for water intake from 16 h before surgery. The analgesia was conducted on the rats, and they remained in the dorsal position, and the loss of recovery reflex or pull-in reflex and respiratory stability were both confirmed just before the chamber attachment and surgery.

### 4.2. Blood Vessel Extraction by U-net

In this subsection, the parameter setting in U-net [23] and the performance evaluation on the blood vessel extraction are described, respectively. The training and extraction of U-net were conducted by a Windows PC with the graphics processing unit (GPU) of Quadro P5000 (NVIDIA Co.) and 16 GB of memory.

#### 4.2.1. Parameter Setting

In the training phase of the U-net model, both the corrected image (RGB) and its vessel enhancement image (gray) were used as input images, and the annotated vessel image (binary) is used as the output image, whose size is 1384 × 1032 (width × height). Overall, 36, 9, and 5 image samples were, respectively, selected for training, verification, and prediction. By balancing the performance and efficiency, the patches with 256 × 256-size were randomly selected from the three kinds of images and were rotated to generate more patch samples for data argumentation. Here, the five-fold cross-validation was conducted. The batch size was set at 16, the loss function was combined by the cross-entropy and the Dice coefficient, and the optimizer was selected as the Adam. By the grid search, the learning rate was determined as 0.001, and the training was completed after $1\times 10^{4}$ iterations. Table 2 lists the parameter settings in U-net training.

#### 4.2.2. Performance Evaluation

**Figure 8:**
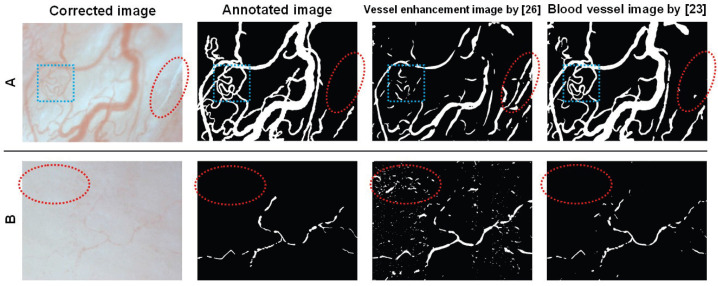
Qualitative comparisons of blood vessel extraction. (**A**) An image sample with sharp vessels. (**B**) An image sample with blurry vessels.

Figure 8 shows the qualitative comparisons of blood vessel extraction in two scenarios. For the image sample with sharp vessels in Figure 8A, the enhancement filtering [26] could not extract those vessels with complex structures; in contrast, the adopted U-net model [23] could well extract them (see cyan rectangle). In addition, some false vessel extractions were found for the enhancement filtering; in contrast, few false extractions occurred for the U-net model (see red circle). For the other image samples with blurry vessels in Figure 8B, more false vessel extractions were found for the enhancement filtering. In contrast, the U-net model still could obtain relatively true extractions (see red circle).

Using 5 image samples, the blood vessel extraction was quantitatively evaluated by the key metrics of accuracy, recall, and specificity, where a large value generally suggests a good performance. Table 3 lists the average extraction performance including the adopted U-net model. Compared with the enhancement filtering, the adopted U-net model obtained higher accuracy of 0.952, which suggested more correct pixel-wise binary classifications. In addition, the larger recall of 0.811 and specificity of 0.978 by the U-net suggested that more vessel fields were extracted and more non-vessel fields were not extracted, as expected. The good extraction performance by the U-net was helpful for the following estimations of blood velocity and vessel diameter.

### 4.3. Results

The selected five rats in each group (sham, CLP, and CLP+TM) have, respectively, independent vital signs measurements and estimations of blood velocity or vessel diameter at each time-step. To better compare the measurements or estimations among the three groups, the measurements or estimations on the five rats in each group were averaged in this subsection.

#### 4.3.1. Vital Signs Measurements on Temporal Changes

Before describing the estimated velocity and vessel diameter of blood, the measurements of four vital signs, namely lactate, SO${}_{2}$, HR, and RR, were explained. Specifically, temporal changes in lactate levels were closely related to sepsis and therapy.

**Figure 9:**
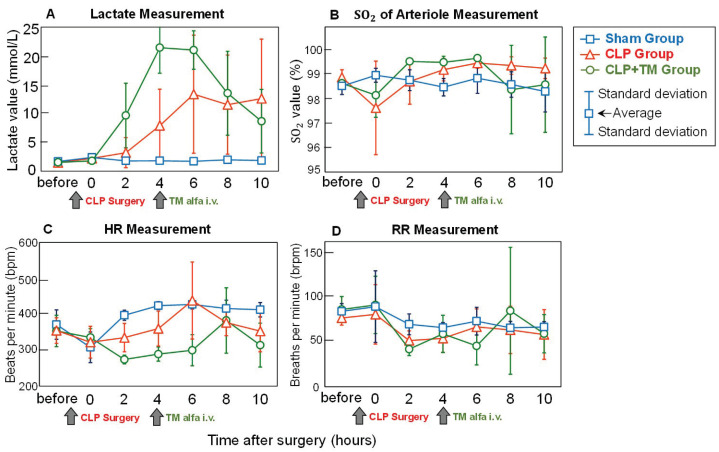
Comparisons of average vital signs measurements. (**A**) Lactate measurement. (**B**) Oxygen saturation (SO${}_{2}$) of arteriole measurement. (**C**) Heart rate (HR) measurement. (**D**) Respiration rate (RR) measurement.

**Lactate Measurement:**Figure 9A shows the measurement of lactate levels before and every two hours after surgery, wherein the average lactate values and plus–minus standard deviations for every five rats in each group are depicted. When a human being diagnosed with septic shock has a lactate value over 2.0 mmol/L, he/she is in a dangerous state [24]. As shown in Figure 9A, for the sham group without CLP surgery, the measured lactate value was always lower than 2.0 mmol/L during the observation period. In contrast, after CLP surgery, the lactate measurement clearly increased over 2.0 mmol/L in the CLP group. In the CLP+TM group, an obvious increase in lactate levels was also found after CLP surgery; subsequently, the measured lactate decreased after the i.v. injection of TM alfa at 4 h after surgery. It is suggested that the temporal changes in lactate measurement for the three groups correlate with the effects of sepsis and therapy. The temporal changes in lactate measurements for the three groups might correlate with the effects of sepsis and therapy, which was consistent with the conclusion in [29], focusing on capillary measurements during sepsis.

**SO${}_{2}$, HR, and RR Measurements:** In addition to the measurement of lactate, we also measured the other three vital signs, SO${}_{2}$, HR, and RR, to investigate the possible correlations between the effects of sepsis and therapy. For both the SO${}_{2}$ and RR measurements, the temporal changes in the three groups (sham, CLP, and CLP+TM) were relatively small, with slight differences among them (Figure 9B,D). Unlike the measurements of SO${}_{2}$ and RR, the temporal changes in HR measurement for the three groups showed marked differences. However, the HR measurement changed markedly even in the sham group, resulting in difficulty in comparison with those for the CLP and CLP+TM groups. Based on the measurement results of SO${}_{2}$, HR, and RR, no evident correlations with the effects of sepsis and therapy were found.

#### 4.3.2. Estimations on Temporal Changes of Blood Velocity and Vessel Diameter

In this subsection, quantitative estimations of the velocity and vessel diameter of blood are described.

**Figure 10:**
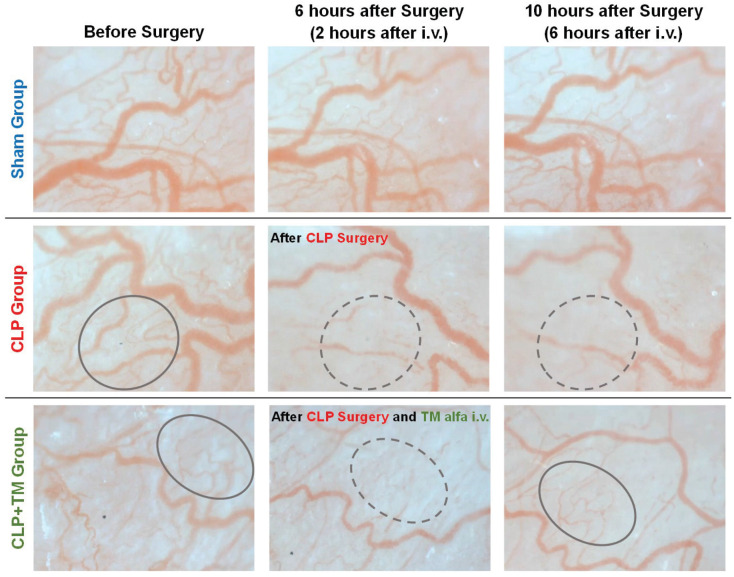
Qualitative temporal changes of microcirculations of the model rats in all the three groups, as shown by the corrected images.

**Blood Velocity Estimation:** The temporal changes in the RBCs flow velocity (i.e., blood velocity) of the model rats can be qualitatively determined by the motion pictures of each panel in Figure 10. For the sham group, we confirmed that the blood velocity did not change significantly by observing the acquired motion pictures. Unlike the sham group, the blood flow in the CLP group slowed from 6 h after CLP surgery, suggesting sepsis-induced microcirculatory dysfunction. Until 6 h after the CLP surgery (just 2 h after the TM alfa i.v. injection), the downtrend of blood velocity of the rats in the CLP+TM group was consistent with that in the CLP group, based on the motion pictures observation. The recovery of blood velocity was observed from 10 h after CLP surgery, namely from 6 h after the injection of TM alfa. The recovery of blood velocity also revealed the effect of TM alfa on sepsis.

**Figure 11:**
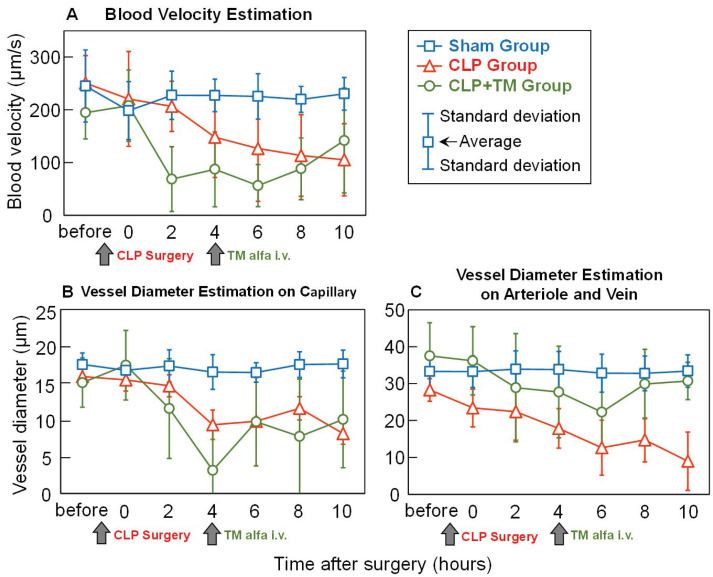
Comparisons of average quantitative estimations. (**A**) Blood velocity estimation. (**B**) Vessel diameter estimation on capillary. (**C**) Vessel diameter estimation on arteriole and vein.

Figure 11A shows the estimation results of blood velocity used to quantitatively evaluate the temporal changes in microcirculation. Here, only the velocity of the arteriole and vein with a diameter between 20 and 100 μm is estimated, considering that the arteriovenous vessels are relatively thick and usually do not disappear even after CLP surgery. In Figure 11A, the average estimations and plus–minus standard deviations of blood velocity for every five rats in each group are depicted before and every 2 h after surgery. Consistent with qualitative temporal changes, the estimated blood velocity in the sham group was relatively steady. In contrast, in the CLP group, the estimated blood velocity continuously decreased after CLP surgery. For the CLP+TM group, the estimated blood velocity also degraded until approximately 4 h, after which it increased owing to the injection of TM alfa. Note that referring to the relatively steady blood velocity change (approximately 150–250 μm/s) in the sham group, before the CLP surgery, the sections of vessels with a close blood velocity were selected for estimating blood velocity change in both CLP and CLP+TM groups.

**Blood Vessel Diameter Estimation:**Figure 10 shows that the temporal change in the capillary diameter smaller than 20 μm is particularly obvious. In the sham group, only a slight variation in the vessel diameter of the capillary was confirmed throughout the experiments. Unlike the sham group, obvious decreases in vessel diameters in the CLP group were observed 6 h after CLP surgery. In particular, the capillaries are narrow in the region indicated by the dashed circles in Figure 10. This tendency suggests sepsis-induced microcirculatory dysfunction. At 6 h after CLP surgery (just 2 h after TM alfa i.v. injection), the vessel diameters of the rats in both the CLP and CLP+TM groups decreased. We found the recovery of vessel diameters at 6 h after the injection of TM alfa (10 h after CLP surgery) in the region indicated by solid circles. The effect of TM alfa on sepsis was also revealed by the recovery of the vessel diameter.

As shown in Figure 11, a vessel diameter of 20 μm is chosen to distinguish blood vessels into two types: one is a capillary whose diameter is less than 20 μm, while the other is an arteriole and a vein, whose diameter is in the range of 20–100 μm. Figure 11B,C show that both the vessel diameter estimations on the two kinds have consistent tendencies of temporal change with those of the blood velocity estimations shown in Figure 11A. In particular, for the CLP+TM group, the recovery of vessel diameters from degradation to 4–6 h after CLP surgery verifies the effectiveness of the injection of TM alfa. Note that referring to the relatively steady vessel diameter change (approximately 10–20 μm for capillary and 20–40 μm for arteriole and vein) in the sham group, before the CLP surgery, the sections of vessels with a close diameter were selected for estimating vessel diameter change in both CLP and CLP+TM groups.

## 5. Discussion

Aiming at the four measurement indices (lactate, SO${}_{2}$, HR, and RR) and the two key estimation indices (blood velocity and vessel diameter), along with normalization with centralization and a significant difference test [30] on the averages of every five rats, temporal changes of typical individual rats, and limitations of the proposed estimation approaches are discussed.

### 5.1. Normalization with Centralization

**Figure 12:**
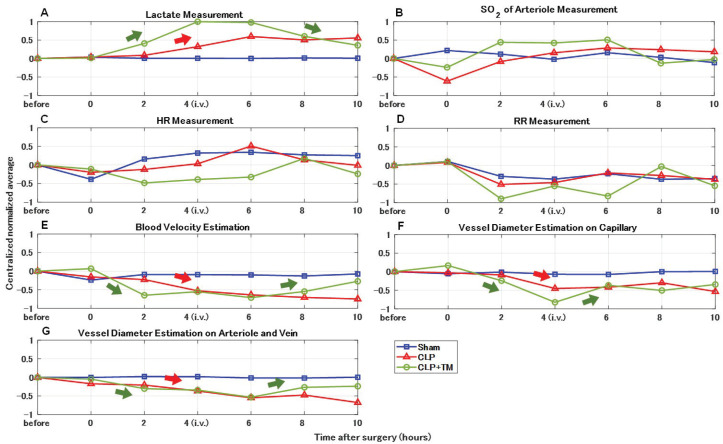
Comparisons of centralized normalized average vital signs measurements and quantitative estimations. (**A**) Lactate measurement. (**B**) Oxygen saturation (SO${}_{2}$) of arteriole measurement. (**C**) Heart rate (HR) measurement. (**D**) Respiration rate (RR) measurement. (**E**) Blood velocity estimation. (**F**) Vessel diameter estimation on capillary. (**G**) Vessel diameter estimation on arteriole and vein.

To better observe the temporal changes in average vital sign measurements and quantitative estimations, the statistical results in Figure 9 and Figure 11 were successively normalized and centralized for all three groups (sham, CLP, and CLP+TM) (see Figure 12). From 0 h after the CLP surgery, the general uptrends of lactate measurement in both the CLP and CLP+TM groups can be found in Figure 12A, and the recovery in the CLP+TM group was observed from 4 h after TM alfa i.v. injection. In addition, the associated variation tendency of the estimations of blood velocity and vessel diameter (capillary or arteriovenous) is shown in Figure 12E–G. In other words, the results in the CLP group presented a general downward after 0 h, and those in the CLP+TM group presented a general U-shaped change whose turning point emerges around 4–6 h. These findings suggested the correlations between the lactate measurement and the estimations of blood velocity and vessel diameter, under the impacts of sepsis and therapy. In contrast, we could not find relatively evident variation tendencies from the measurements of SO${}_{2}$, HR, and RR (see Figure 12B–D), which might not be evidently affected by the sepsis-induced coagulation.

### 5.2. Significant Difference Test

**Figure 13:**
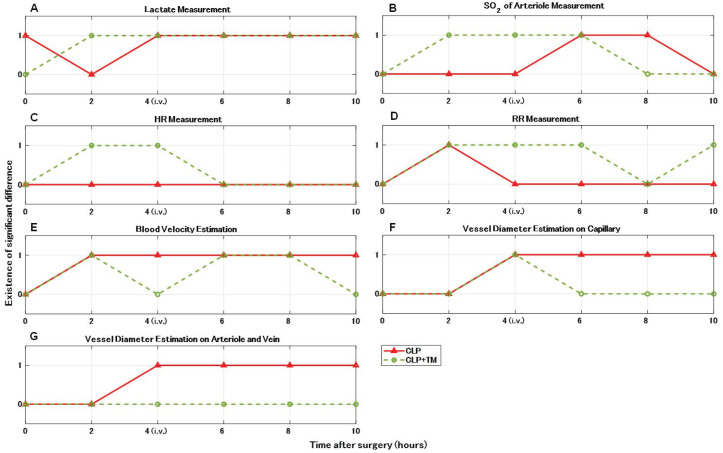
Results of significant difference test on vital signs measurements and quantitative estimations compared with those before cecum ligation and puncture (CLP) surgery (α = 0.1). (**A**) Lactate measurement. (**B**) Oxygen saturation (SO${}_{2}$) of arteriole measurement. (**C**) Heart rate (HR) measurement. (**D**) Respiration rate (RR) measurement. (**E**) Blood velocity estimation. (**F**) Vessel diameter estimation on capillary. (**G**) Vessel diameter estimation on arteriole and vein.

Using the measurement and estimation results of all the five rates both in the CLP and CLP+TM groups, we performed the Student’s *t*-test [30] on each group every two hours after surgery to investigate whether and when significant differences occur for each index in comparison to the measurements and estimations before surgeries. Figure 13 shows the results of the significant difference test (0: non-existence, 1: existence) for the four measurement indices and the three estimation indices, in which the significance level α is set to common 0.1.

For the CLP group, significant differences occurred in most indices except for the HR measurement shown in Figure 13C, showing successive occurrences in the lactate measurement and the estimations of blood velocity and vessel diameter (capillary or arteriovenous) after 2 h (see Figure 13A,E–G). In particular, compared with the start time of successive occurrences of significant differences in the lactate measurement and vessel diameter estimations, namely 4 h, that (2 h) of the blood velocity estimation is earlier. Thus, the correlations of lactate measurement and the estimations of blood velocity and vessel diameter might be associated with sepsis. Specifically, blood velocity estimation could show an earlier correlation.

Considering the results of the significant difference test for the CLP group, we focused on the significant differences in lactate measurement and the estimations of blood velocity and vessel diameter for the CLP+TM group. Among the focused four indices shown in Figure 13A,E–G, the occurrence (at 4 h) and successive disappearance (after 6 h) of significant differences were found in the vessel diameter estimation on the capillary, as shown in Figure 13F. The change in capillary diameter could reflect the effect of sepsis therapy.

### 5.3. Typical Individual Rats

**Figure 14:**
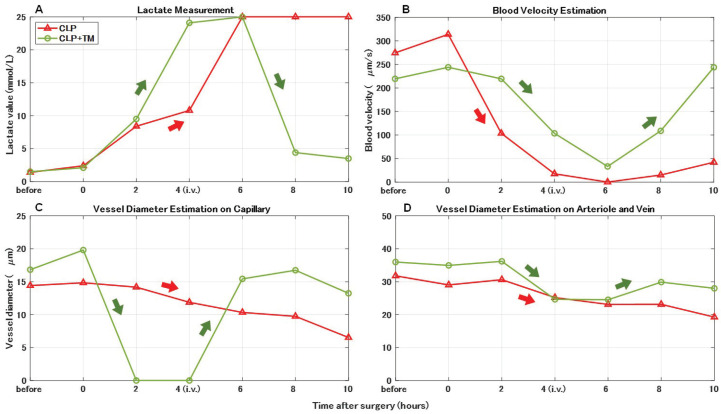
Comparisons of vital signs measurements and quantitative estimations of each typical rat. (**A**) Lactate measurement. (**B**) Blood velocity estimation. (**C**) Vessel diameter estimation on capillary. (**D**) Vessel diameter estimation on arteriole and vein.

Figure 14 shows the temporal changes in lactate measurement and the estimations of blood velocity and vessel diameter (capillary or arteriovenous) in some typical individual rats. Compared with the average results of the five rats in Figure 9A and Figure 11, the statistical results of each typical rat represent more obvious variation tendencies, as shown in Figure 14. More concretely, for the CLP group, the lactate measurement value markedly increased after CLP surgery (see Figure 14A), while the estimations of blood velocity and vessel diameter contrarily decreased (see Figure 14B–D). For the CLP+TM group, the tendencies of temporal changes in all the results reversed after 4–6 h owing to the TM alfa i.v. injection, suggesting its effects on sepsis.

### 5.4. Limitations

In the RPCA-based decomposition of the corrected image (see Figure 2), the sparse image generated with RBCs contained certain noises, which were unavoidable due to the respiration and tissue motions of model rats. Unavoidable noise might lead to errors when the proposed blood velocity estimation approach is used. In other words, the performance of the blood velocity estimation is affected by the noise strength in the corrected image, which originates from the acquired image of motion pictures.

Meanwhile, although the polarization plate was utilized to reduce the specular reflection caused by the rat tissue surface in the non-contact imaging setup, the residual specular reflection might blur some fields of the acquired images (see Figure 1C). Due to the blurred fields in the acquired images, microcirculatory information might be corrupted or lost, resulting in difficulties in the analysis of microcirculation images.

## 6. Conclusions and Future Work

This study aims at providing some findings on the microcirculation of septic rats and TM alfa-treated rats both qualitatively and quantitatively. These findings should become basic knowledge in physiology and medicine. The whole system developed here might be useful for pharmaceutical companies to confirm the effect of the medicine on an image basis. Furthermore, the proposed image analysis method could be applied to some commercial products.

To analyze the microcirculatory images of the model rats with different operations, including CLP and TM alfa injection, we proposed two estimation approaches for key indices of microcirculation, namely blood velocity and blood vessel diameter. Based on our proposal, the decrease in blood velocity and blood vessel diameter in the CLP group and the recovery after the decrease in those in the CLP+TM group were quantitatively estimated.

Due to the noise from the respiration and tissue motions of model rats and the specular reflection of the imaging setup, the proposed estimation approach could not acquire an exact blood velocity. Thus, the corresponding noise elimination may be a future research direction. In addition, to further assess microcirculation, more helpful indices can be estimated, such as vessel density and complexity, and heart rate.

## Figures and Tables

**Table 1:** Properties of septic model rats.

Property	Specification
Breed	Wistar
Age	12 weeks
Gender	Male
Body weight	240–290 g
Amount of rats	15
Number of rats in each group	5
Group names	(1) Sham;
	(2) CLP;
	(3) CLP+TM

**Table 2:** Parameter setting in U-net [23].

Property	Specification
Image size (width×height)	1384 × 1032 pixel
No. of images	36:9:5
(training:verif.:prediction)	
No. of channels in input image	4 (RGB + gray)
No. of channels in output image	1 (binary)
Patch size	256 × 256 pixel
Batch size	16
Loss function	Cross-entropy+Dice
Optimizer	Adam
Learning rate	0.001
No. of iterations	$1\times 10^{4}$

**Table 3:** Performance evaluations on blood vessel extraction.

	Accuracy	Recall	Specificity
**Enhancement filtering [26]**	0.130	0.529	0.030
**Adopted U-net [23]**	0.952	0.811	0.978

## Data Availability

Not applicable.
